# Ciprofloxacin-Collagen-Based Materials with Potential Oral Surgical Applications

**DOI:** 10.3390/polym12091915

**Published:** 2020-08-25

**Authors:** Daniel-Cristian Ioan, Ileana Rău, Mădălina Georgiana Albu Kaya, Nicoleta Radu, Marinela Bostan, Roxana Gabriela Zgârian, Graţiela Teodora Tihan, Cristina-Elena Dinu-Pîrvu, Alina Lupuliasa, Mihaela Violeta Ghica

**Affiliations:** 1 Department of General Chemistry, Faculty of Applied Chemistry and Materials Science, University POLITEHNICA of Bucharest, 1-7 Gh. Polizu Str., 011061 Bucharest, Romania; cristi_wol@yahoo.com (D.-C.I.); zgirianroxana@yahoo.com (R.G.Z.); gratielatihan@yahoo.com (G.T.T.); 2 Department of Collagen, National Research and Development Institute for Textile and Leather (INCDTP), Division Leather and Footwear Research Institute, 93 Ion Minulescu Str., 031215 Bucharest, Romania; albu_mada@yahoo.com; 3 Faculty of Biotechnology, University of Agronomic Sciences and Veterinary Medicine of Bucharest, 54 Marasti Boulevard, 011464 Bucharest, Romania; nicolbiotec@yahoo.com; 4 Biotechnology Department, National Institute for Chemistry and Petrochemistry R&D of Bucharest, 202 Splaiul Independentei, 060021 Bucharest, Romania; 5 Institute of Virology Stefan S. Nicolau, Immunology Centre, 285 Mihai Bravu Avenue, 030304 Bucharest, Romania; marinela.bostan@yahoo.com; 6 Department of Physical and Colloidal Chemistry, Faculty of Pharmacy, University of Medicine and Pharmacy “Carol Davila”, 6 Traian Vuia Str., 020956 Bucharest, Romania; ecristinaparvu@yahoo.com (C.-E.D.-P.); alina.lupuliasa@drd.umfcd.ro (A.L.); mihaela.ghica@umfcd.ro (M.V.G.)

**Keywords:** ciprofloxacin, collagen-based materials, cytotoxicity, drug release

## Abstract

We report in this paper the synthesis and characterization of a new collagen-based material. This material was obtained in a spongy form and was functionalized with an antibiotic, ciprofloxacin. The targeted applications of these kind of materials concern the post-operative prophylaxis. The in vitro tests (antimicrobial, cytotoxic, drug release) showed that sponges with a concentration of 0.75 g of ciprofloxacin per gram of collagen could be beneficial for the desired applications.

## 1. Introduction

In head and neck surgery, bone and skin grafts can be used for the reconstruction of the maxillary or mandible, as well as for the oral mucosa or the hard palate [1,2]. Bone grafts can be harvested from the fibula, ribs, or iliac crest for reconstruction [3,4,5]. Before the operation closes and to prevent local infections different antibiotics materials can be topically applied. The same rules apply to tegument grafts when skin grafts from the thigh or arm are used for reconstruction of the oral mucosa in the oral cavity or to muscle repositioning surgery (e.g., repositioning of the sternocleidomastoid muscle at the level of the oral floor to achieve the swallowing function as much as physiological possible) [6]. Various other operations such as small oral extractions in certain diseases, such as hemangioma of the lips or tongue, could have an improved wound healing and prophylactic protection due to the antibiotic [7]. In other cases, these antibiotic-based materials can be used in trauma, such as fractures of the facial mass, maxillary or mandible, zones that could be potentially already infected areas. Another important aspect is represented by the possible hemostatic role of these materials knowing that both the head and the neck are intensely vascularized anatomical areas. Also, such materials can be used for dental extractions, taking into account that the alveolar vessels with a very high bleeding potential represents an area where hemostasis can hardly be achieved (usually with a gelaspon sponge, hemostatic powder—arista, here, being inefficient at the electrocautery).

Therefore before, during, or after surgery there may be infections or superinfections. Preoperative, in most cases, are traumatic factors or superinfected formations not treated in time which determine infections while during surgery there may be pathogenic factors by contamination of the wound. Post-operative superinfection may occur due to poor local hygiene and in some rare cases there may be a negative or allergic reaction of the body or there may be seromas (fluid collection at the suture) that can be superinfected by accumulating purulent fluid. Superinfections with purulent fluid are the most common complications, especially in case of poor local hygiene.

The preventive effect of the routine use of preoperative surgical antibiotic prophylaxis on the occurrence of surgical site infections prior to non-clean surgery such as different types of implant surgery or different types of grafting in surgery has been long time recognized, but the benefit of continued surgical antibiotic prophylaxis after completion of the procedure is still unclear. A large amount of worldwide evidence shows that a single preoperative dose of surgical antibiotic prophylaxis may be not inferior to additional post-operative multiple doses for the prevention of surgical site infections including intraoperative doses according to the duration of the surgical intervention [8,9,10].

Antibiotics are a very important adjuvant treatment in the infectious invasive pathologies, and, therefore, usually, the prevention of surgical wound, implants or various types of grafting related infections in the operating treatment is made by prophylactic administration of antibiotics. It should be underlined that the antibiotics adjuvant treatment is effective only with a complete and good surgical treatment of the infection site. In addition, the implants are avascular materials that cannot be cured simply with oral drugs, the antibiotics may reach them only by diffusion from surrounding tissues [11]. 

Antisepsis has been contributing in a major way in lowering post-operative complications, morbidity, and mortality. There are a few worldwide principles for antibiotics, for example the duration of administration of the prophylactic antibiotic that should be as short as possible but long enough till necessary or the pathogenic spectrum of antibiotics and, in this respect, we are looking for antibiotics effective against as many as possible potential pathogens [12].

Compared to the systemic therapy the local antibiotic therapy has several advantages, the benefits including high and sustained concentrations at the site of infection where local physiological changes may hinder the efficacy of systemic antibiotics. On the other hand, local hypersensitivity or reactions related to contact dermatitis and interference with local wound healing can be problematic, a major disadvantage of local antibiotics is that there are no specific efficacy criteria to be standardized or approved by any official agency. [13].

Local antibiotics are typically not applied until just before closure, where some may suggest that there is a possibility that they would limit their use other than an adjunct drug to parenteral prophylaxis. There is evidence to suggest that there is a finite period during which prophylactic antibiotics may suppress an incisional infection. One of the most important attraction of local or topical antibiotics is represented by the low systemic toxicity. Surgical guidelines recommend antibiotic prophylaxis in different kind of surgical procedures, especially in cases with various types of implants or prosthesis or various types of grafting [14,15,16,17]. It was proved that the incidence of surgical wound infections is reduced when antibiotic prophylaxis is administered, usually prophylaxis being normally recommended for all clean–contaminated procedures. Also, for clean procedures, this prophylaxis may be considered necessary for certain patients and various types of surgery that meet specific risk criteria [18].

Ciprofloxacin is an antibiotic based on fluoroquinolone and generally used in bacterial infections such as skin infections, bone and joint infection, urinary tract infections, or respiratory infections. There are some studies devoted to materials based on ciprofloxacin [19,20,21,22,23,24]. M. Suhaeri et al. [19] proposed a novel skin patch that combines antibiotic, cell-derived extracellular matrix (ECM) and biocompatible polyvinyl alcohol (PVA) hydrogel for infected wound healing, while F. Puoci et al. [20], R. Sripriya et al. [21], F. Lukitowati et al. [22] and K. Shanmugam et al. [23] used as matrix for the antibiotic the collagen and proposed different types of materials. An interesting study devoted to ciprofloxacin released from different materials was performed by M. T. Arafat et al. [24]. In these studies, the prepared materials showed efficiency.

Despite the fact that ciprofloxacin could have as side effect tendon disorders, with the risk of tendinopathy [25,26,27] we have selected ciprofloxacin due to its ability to replace other antibiotics being an alternative for allergic patients at other types of antibiotics used in head and neck surgery.

In the past decade, many studies were devoted to natural-based materials [28,29] and particularly to collagen-based materials [30,31,32,33,34,35,36] with biomedical applications. This was due to the proved polymer biocompatibility with the human tissues. Different studies showed that collagen-based materials could be functionalized with different types of drugs such as anti-inflammatory or antibiotics. The adequate physical-chemical, biopharmaceutical and biological characteristics showed that these materials could be used for burns or in head and neck surgical applications. The present study is devoted to the synthesis and characterization of a spongy collagen-based material with ciprofloxacin in view of head and neck surgery antibiotic prophylaxis, these collagen-based sponges with ciprofloxacin, being beneficial for the wound management due to both the properties of collagen and antibiotic. In this respect the sponges were tested against *Escherichia coli* as gram negative bacteria, *Staphylococcous aureus* as gram positive bacteria, and *Candida albicans* and *Candida parapsilopsis* as fungus. These microorganisms were chosen because it has been statistically demonstrated that these microorganisms are usually involved in the head and neck surgery infections [37]. For example, *S. aureus*, a pathogen included in the spectrum of ciprofloxacin as many other bacteria, may affect the regions of the head and neck, such as oral infections with various symptoms such as redness and swelling of the mouth, or burning sensations. This type of bacteria may also be found in dental abscesses. Another sensitive region of the head and neck for infections may be the eye where the eyelid that serves to protect the ocular surface and the glands could be easily infected with S. aureus. At the same time, another potential site for infection with *S. aureus* is the nose cavity where it may cause even crusts and bleeding, collagen-based ciprofloxacin sponges being able to help in this types of cases through the properties of the antibiotic and the hemostatic and regenerative properties of the collagen, these features representing a true benefit in other types of surgery. 

Taking into consideration that these materials are envisaged for head and neck surgery cytotoxicity tests were performed using human umbilical vein endothelial cells (HUVEC) knowing their properties to mimic human normal cells organism reactions to various drugs. The HUVEC cells line is often used in comparative studies aimed to evaluate in vitro the effect of different therapeutic schemes on both normal cells and tumor cells [38,39], in order to quantify their cytotoxicity on human healthy cells. Moreover, they are used in laboratories as model systems for studying the function and pathology of endothelial cells. At the same time, they provide a classic model system to study many aspects of the organism regarding various types of disease, tumors, inflammation or other pathological conditions 

## 2. Materials and Methods 

### 2.1. Materials

Collagen-based sponges were prepared starting from a collagen gel of 1.91% and acidic pH. This gel was obtained from bovine derma using a current technology at INCDTP—Division Leather and Footwear Research Institute, Collagen Department (Bucharest, Romania) [40,41]. Ciprofloxacin Hydrochloride (MP Biomedicals, LCC, Solon, OH, USA) was added in concentration of 0.5; 0.75 and 1 g/g to a collagen solution (1%, pH 7.4) prepared from the initial gel with 1 M sodium hydroxide. The obtained solutions were further cross-linked with 0.5% glutaraldehyde reported to dry collagen. The sponge materials were obtained by lyophilization process consisting of three steps: (i) freezing at −55 °C for 1 min; (ii) drying at −55 °C for 15 h—time in which approximately 90% of water is sublimated; and (iii) final-drying at −40 °C for 10 min. The sublimation process took place under vacuum and was performed using Delta 2-24 LSC (Martin Christ, Osterode am Harz, Germany) equipment. All obtained collagen sponges of 2 cm diameter and 0.5 cm thickness were packed in polyethylene bags and sterilized at 254 nm using Vilber–Lourmat equipment (Suebia, Germany).

### 2.2. Water Absorption 

Collagen-based sponges of 1 cm × 1 cm × 0.5 cm were immersed at 25 °C in distilled water to evaluate the water absorption capacity. At different time periods the water absorption was calculated using Equation (1). Every test was performed in duplicate.

(1) $$\%\;water\;absorption=\;\frac{w_{1}-w_{0}}{w_{0}}\times 100$$

where *w*_0_ is the dry mass and *w*_1_ is the wet mass.

### 2.3. Enzymatic Degradation

The behavior against collagenase (obtained from *Clostridium histolyticum*), supplied by Sigma-Aldrich (Saint Louis, MO, USA) was studied using phosphate buffer solution (PBS) with pH 7.4. Samples of a specific weight were immersed in PBS solution containing collagenase (10 μg/mL) and incubated at a temperature of 37 °C. At different time intervals the samples were weighed, and the mass loss was estimated according to Equation (2). Each biodegradation test was performed in duplicate.

(2) $$\%\;collagen\;mass\;degraded=\;\frac{w_{i}-w_{t}}{w_{i}}\times 100$$

where *w_i_* is the initial mass and *w_t_* is the mass after time *t*.

### 2.4. In Vitro Ciprofloxacin Release

The in vitro release of ciprofloxacin from the prepared materials was conducted using a sandwich device adapted to a paddle dissolution equipment, as previously described [30]. The concentration of the drug presented in each aliquot, collected from the release medium at predetermined time points, was spectrophotometrically evaluated at its maximum absorbance corresponding to a wavelength of 271 nm and using the standard calibration curve. 

The cumulative drug percentage released was then assessed. The kinetic data were fitted with several models: the Power law model (Equation (3)) and its particular cases, Higuchi (n = 0.5) and Zero-Order (n = 1). Based on the fitting results the drug release mechanism was conceived.

(3) $$\frac{m_{t}}{m_{\infty }}=k\times t^{n}$$

where *m_t_/m*_∞_ is the fractional release of drug at time *t*, *k*—the kinetic constant, *n*—the release exponent indicating the drug release mechanism. The release experiments were carried out in triplicate.

### 2.5. In Vitro Biological Effects of Ciprofloxacin-Collagen Sponges on Some Pathogen Microorganism

In vitro tests on pathogen microorganisms were performed using as gram negative bacteria *Escherichia coli* ATCC 10536, as gram positive bacteria—*Staphylococcous aureus* ATCC 6538, and as fungus—*Candida albicans* ATTC 10231 and *Candida parapsilopsis* ATCC 22019. The inoculation has been realized with suspended microorganisms in physiological serum, according to the McFarland 0.5 standard.

The tested bacteria (gram negative or gram positive) were cultivated on Luria Bertani (DSMZ 381) culture medium while the fungi (*Candida* sp.) were cultivated on PGA culture medium (potato glucose agar.). All the experiments were conducted in triplicate, in Petri plates of 50 mm, in a microbiological hood with laminar flow.

Two solutions of 1000 µg/mL and 100 µg/mL of ciprofloxacin in physiological serum were prepared and sterile papers of 0.4 cm diameter were impregnated with these solutions. Pieces of collagen-based material, with the same weight, were obtained by cutting the initial sponges with a sterile scissor, in aseptic conditions. Each sample (collagen-based piece or ciprofloxacin paper) was then introduced in a Petri plate inoculated with the studied microorganisms.

### 2.6. Cytotoxic Study of Collagen-Based Materials on Normal Cells—In Vitro Tests

As normal cells the HUVEC line cell (ATTC PCS 100-010) was used and as culture medium Endothelial Cell Growth (ATCC PCS100041). For the tests, a microbiological hood with laminar flow and an Elisa EZ 400 Biochrom analyzer were used.

The collagen-based materials with identical mass were introduced in the culture mediums of Vascular Cell Basal Medium (ATCC PCS–100030) with HUVEC viable cells. After 6 h, 24 h, and respectively 48 h, the solid materials were pull out from de mediums, and the numbers of viable cells were quantified using the MTT method [38,42,43,44,45]. All determinations have been done in plates of 96 wells, in triplicate, each determination being performed for 3 times. A sample of HUVEC untreated cells was used as witness. The untreated cells growth has quantified using the same methodology.

The effect quantification was performed through the proliferation index (PI) determination Equation (4).

(4) $$PI=\;\frac{OD\;of\;treated\;cells}{OD\;of\;untreated\;cells\;\left(witness\right)}$$

where *OD* is the optical density.

### 2.7. Statistical Analysis

All the experiments performed on the collagen materials were carried out in duplicate or triplicate, and the data obtained were analyzed for statistical significance. Error bars reported within the charts denote the standard errors of the mean. The results were reported as samples mean ± standard deviation (SD) of independent replicates.

## 3. Results and Discussion

### 3.1. Water Absorption

Important feature of such spongy materials is given by their capacity to absorb water. When the collagen-based sponge with ciprofloxacin is inserted/applied in the wound during surgery, a synergistic effect occurs through the simultaneous action of the surrounding tissue. At the buccal level, depending on the region, through blood or through blood and saliva, a local hemostasis could take place and local external factors of the body contribute to the degradation/disintegration of the sponge, which, at the end, is absorbed by the body. At the level of other wounds this kind of sponge is specifically indicated for its resorption capacity being the main reason for the use of collagen sponges in venous or capillary hemorrhages, for diffuse bleeding, for hemostasis in case of dental extractions, or for burns of 2nd or 3rd degree. At the time of resorption of the collagen sponge the drug is released and the ciprofloxacin is assimilated locally by neighboring tissues and being in much lower quantities than the amounts of antibiotic administered orally or intravenous, the potential systemic complications are reduced. Thus, the absorption capacity is also related to drug release from sponges as drug diffusion depends on fluid penetration in the porous structure [33]. Compared to absorbable sutures that can be resorbed 7-90-120 days after use, the collagen-based sponge is resorbed much faster in 24–48 h or a maximum of 96 h, being absorbed much faster than the gelatin-based sponge (gelaspon) that reabsorbs in 3–4 weeks of use. Collagen-based sponges are much faster assimilated by the body due to the synergistic effect of local factors (blood, lymph, saliva in the oral cavity).

**Figure 1:**
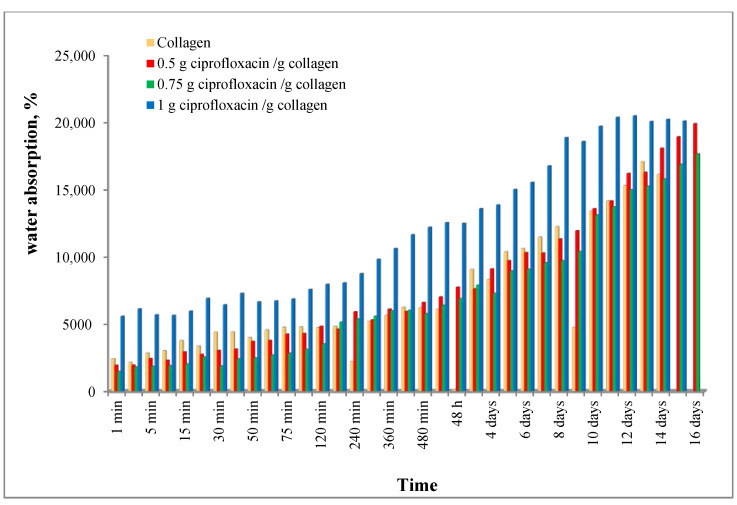
Water absorption behavior for the studied samples.

Figure 1 presents the water absorption capacity of the synthetized materials. It can be seen that in time, the water quantity absorbed is increasing for all samples, but the water up-take is depending on the drug concentration. For samples with smaller drug concentration (0.50% and 0.75%) the water absorption is similar, even smaller, to that of collagen sponges (without drug). For the sponges with the highest drug concentration studied this water up-take is higher at all the times, this behavior being due to probably the drug which absorb more water than collagen.

The high retention of fluid in the porous structure indicates that a large amount of biological fluid can be absorbed when such supports are in contact with a wound during surgery, adhering and favoring the formation of new regenerated tissue.

### 3.2. Enzymatic Degradation

The collagen-based sponge degradation represents another important feature as this material, at the end, should be resorbable by the body. On the other hand, a faster sponge erosion determines a rapid drug release, while a slow degradation rate conducts to a small drug release, with as consequence an inefficient antibiotic level at the wound. The biodegradation capacity of the designed collagen spongy matrices was in vitro simulated using collagenase solution. The enzymatic degradation of collagen materials is influenced by its triple helical integrity, the cross-linking degree and the availability of cleavage sites [33].

**Figure 2:**
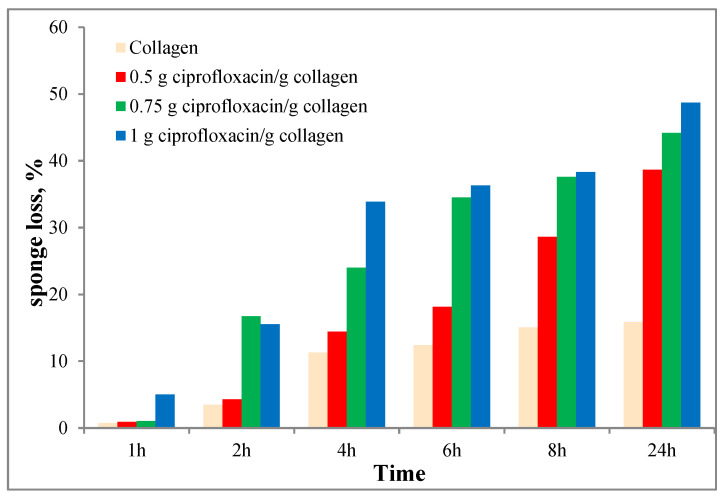
Enzymatic degradation behavior for the studied materials.

In Figure 2 the behavior of the studied sponges concerning the enzymatic degradation is plotted. As expected, this degradation is increasing in time and the ciprofloxacin presence favors the degradation. Thus, it can be seen that the sponges containing ciprofloxacin are almost 50% degraded after 24 h, compared to collagen sponge (approx. 15% degraded). After 24 h the ciprofloxacin sponges disintegrated.

The in vitro biodegradation results showed that a balanced degradation rate is obtained, targeted to get an adequate drug release with direct consequences on treatment efficiency and improved patients compliance.

### 3.3. In Vitro Ciprofloxacin Release

A critical parameter to be considered for the formulation and evaluation of spongy collagen-based material with ciprofloxacin, designed to prevent and control the infection associated with head and neck surgery, is the drug kinetics release, monitored through the influence of drug content on kinetic patterns.

The fraction of ciprofloxacin released at each time point was computed as percentage of the total drug from collagen sponge.

**Figure 3:**
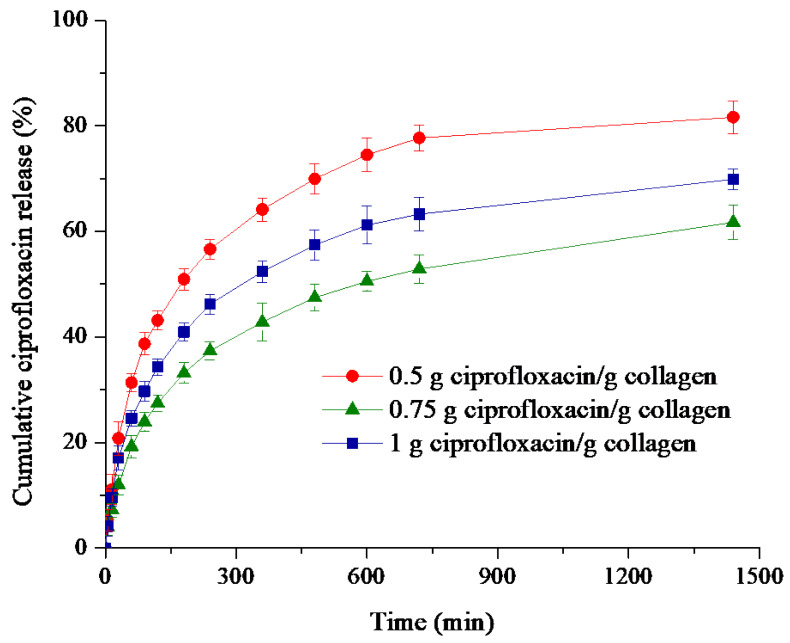
Time-dependent cumulative release profiles of ciprofloxacin from collagen matrices.

Figure 3 illustrates similar kinetic profiles for all three formulations characterized by an initial burst release in the first 2 h followed by a progressive and prolonged drug release for a longer period up to 24 h. 

The sponge with 0.50 g ciprofloxacin/g collagen exhibited a rapid release (43.15%), followed by the sponge with maximum level of ciprofloxacin (34.26%), while the formulation with medium drug concentration leads to the smallest burst effect (27.45%). It can be remarked that release is 1.6 times faster for ciprofloxacin concentration of 0.5% in comparison with 0.75%. The quite pronounced burst release effect could be due to the large sponges water absorption in the first 120 min.

The cumulative antibiotic released percentage after 24 h is of 81.63% for the sample containing 0.50% ciprofloxacin, while the collagen supports having a higher drug content showed 61.70%, respectively 69.86% of ciprofloxacin release within the same period of time (Table 1).

An important aspect to highlight is that during the in vitro release experiments, for each sampling time the released antibiotic concentration was higher than is the one reported for the minimum inhibitory concentration (MIC) for pathogens bacteria such as *Staphylococcus aureus* and *Escherichia coli* [46,47,48,49].

Concerning the data reported by other authors, R. Sripriya et al. [21] described that a ciprofloxacin-loaded collagen bilayer dressing without PVP presented a pronounced drug release after 5 h of experiments (about 78.56%), followed by an integral release after 8 h, while for the same dressing with drug dispersed in PVP, the burst release effect was markedly reduced, the antibiotic being progressively delivered, about 24.45% in the first day, followed by 31.45% and 35.36% in the next 2 and 3 days respectively.

M. Suhaeri et al. [19] reported that a novel skin patch based on ciprofloxacin antibiotic, cell-derived extracellular matrix and PVA hydrogel showed a fast release of antibiotic, the drug being almost completely delivered within 2 h of experiments.

A. Gauzit Amiel et al. [50] showed that some sponges designed for diabetic foot infections, based on chitosan and cyclodextrin polymer in different ratios and impregnated with ciprofloxacin, thermal or non-thermal treated, recorded either a drug rapid release higher than 80% in the first hour and reached a plateau after 4.5 h, or a slower and gradual release, 80% of antibiotic being delivered in 5–10 h.

In another study, M.T. Arafat et al. [24] reported that ciprofloxacin release from collagen fibers varies from about 20–70% in the first hour, to 40–90% drug delivered in the next 7 h, depending on the in situ cross-linking method during fiber wet spinning.

Taking into account the above results, we appreciate that our designed collagen-ciprofloxacin sponges well balanced the burst release effect with gradual and progressive drug release over a long period of time.

Even if the faster release is often avoided and leading to high drug doses reached in the initial step of release profile, for antibiotic delivery the burst effect followed by a gradual release is a beneficial pattern for head and neck surgery. Although the burst release, induced by the drug not entrapped in the sponges matrix, is providing the drug active levels in a short period of time for eradicating and preventing the spreading of harmful bacteria encountered during surgery, the gradual and sustained release, obtained after wound exudate penetration in sponges matrix structure, is securing the avoidance of bacteria regrowth and offering also a prophylactic effect during the long lasting period of wound healing. The results obtained suggest that the sponge with 0.75% ciprofloxacin better equilibrate the burst release effect with the prolonged antibiotic delivery. 

The ciprofloxacin release patterns were further evaluated to set up the kinetic mechanism by fitting the in vitro experimental data with Peppas law model (Equation (3) and its particular cases, Higuchi (n = 0.5) and Zero-Order (n = 1), the corresponding correlation coefficients values being listed in Table 1.

The values of the correlation coefficients R ranging between 0.9762 and 0.9875 indicate a better fitting of Power law model and demonstrate that such model could be used to describe the ciprofloxacin release mechanism from collagen sponges. Moreover, the release exponent values varying from 0.33 to 0.37 reveal a non-Fickian drug transport mechanism which involves many stages: (i) an initial desorption of the drug retained at sponge surface, (ii) absorption of the release medium in the porous structure, polymer hydration and sponge swelling, these two steps corresponding to the burst release effect, followed by the (iii) diffusion of the drug retained in the polymer network during the lyophilization process simultaneously with progressive degradation of the release support, this step being correlated with the prolonged and sustained drug release.

### 3.4. In Vitro Biological Effects of Ciprofloxacin–Collagen Sponges on Some Pathogen Microorganism

Table 2 presents the results obtained for collagen-based materials tested against different pathogens. From this table it can be seen that for *Escherichia coli* bacteria the best results were obtained for the sponge containing 1g ciprofloxacin/g collagen, the result being similar to that obtained for solutions of 1000 g/mL ciprofloxacin. Similar results were obtained for the other ciprofloxacin–collagen materials revealing that E. coli is sensitive to the tested products.

**Figure 4:**
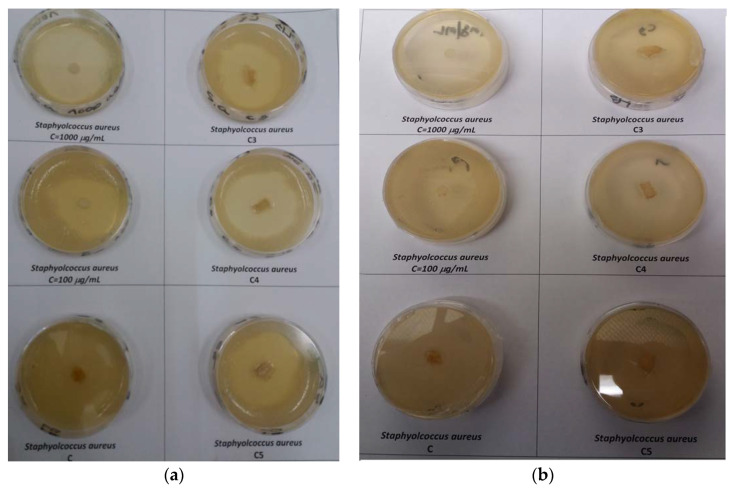
The effect of ciprofloxacin-collagen-based materials on *Staphylococcus Aureus*. (**a**) frontal view and (**b**) dorsal view—for sample code see Table 2.

Important results (Figure 4) were obtained when the synthetized materials were tested against *Staphylococcus aureus*. It is known that ciprofloxacin is not an antibiotic with activity on gram positive microorganisms. Nevertheless the studied microorganism is sensitive to the active substance, the best result being obtained for the sample of 1 g ciprofloxacin/g ciprofloxacin (diameter of inhibition of 44.5 ± 2.22 mm), the diameter of inhibition being bigger than the one observed for the 100 g/mL ciprofloxacin. The results obtained during this study confirm that the mechanism of active substance (ciprofloxacin) is the inhibition of DNA synthesis. The positive results obtained in the case of gram positive microorganism (*Staphylococcus aureus*) can be due to the fact that the research was done with microbials strains from standardized collections, without mutation that may impregnate resistance to the tested antibiotic. These results are similar to those obtained by F. Puoci et al. [20] highlighting the fact that collagen-based materials with ciprofloxacin could be used for the desired applications.

Concerning the fungi type of *Candida* sp. we concluded that none of the studied materials has effect, respectively the ciprofloxacin solution with concentrations of 100 g/mL and 1000 g/mL and neither the collagen-based sponges, this microorganisms being resistant to the active tested substance (ciprofloxacin).

### 3.5. Cytotoxic Study of Collagen-Based Materials on Normal Cells—In Vitro Tests 

To mimic the conditions from medical practice, particularly head and neck surgery, and taking into consideration that the water up-take, drug release and the enzymatic degradation measurements revealed that in 24 h the collagen-based sponges absorb a lot of water, delivered over 80% of antibiotic, and disintegrate, the cells proliferation was determined at 6 h, 24 h, and 48 h. 

Table 3 summarizes the results related to the PI of the tested cells under the ciprofloxacin–collagen sponges exposure. 

The results revealed that in case of the cell line tested after 6 h and 24 h of exposure a PI higher than 1 is obtained suggesting that the cells are stimulated by the sponges. After 48 h of exposure the PI is decreasing and becomes closer to 1 when the cells were treated with ciprofloxacin-based materials. Moreover, in the case of the sample containing 1g ciprofloxacin/g collagen at 48 h the PI decrease was significant (PI = 0.77) showing that this sponge become cytotoxic if it is used more than 24 h for these cells.

## 4. Conclusions

The present study revealed that ciprofloxacin–collagen-based materials under sponge form could be synthetized. The results obtained suggest that even such sponges absorb important quantity of water, these materials could be used in head and neck surgery as they can degrade quite fast about 40–50% during the first 24 h. Moreover, for the same time range the cumulative ciprofloxacin released percentage was between 61.70–81.63%, the kinetic profiles showing a biphasic allure targeting to prevent and control the local infection associated with head and neck surgery, avoiding further bacterial invasion or proliferation.

On the other side these materials revealed bacteriostatic effect, by inhibiting the development of the gram negative bacteria such as *Escherichia coli* and gram positive bacteria such as *Staphylococcus aureus* (*Staphylococcus* sp. of international commercial collection, which does not manifest the phenomenon of chemoresistance). 

Other important finding is that these materials present even a stimulating effect on normal HUVEC cell line and thus they do not present cytotoxic effect for exposure times less than 24 h. Due to the fact that the sponges with 1g of ciprofloxacin/g collagen show a cytotoxic effect on the HUVEC cell line after 24 h exposure it can be concluded that in vitro an optimum effect (antimicrobial and not cytotoxic) is obtained for the sponges with 0.50 g ciprofloxacin/g collagen and 0.75 g ciprofloxacin/g collagen. Corroborating this aspect with the drug release experiment and the physical-chemical results a collagen sponge containing 0.75 g ciprofloxacin for 1 g of collagen could be recommended for in vivo tests in order to confirm the findings presented in this paper.

## Figures and Tables

**Table 1:** Correlation coefficients (R) for Higuchi, Zero-order, and Power law kinetic models; kinetic parameters specific to the Power law model and cumulative ciprofloxacine released percentage.

Ciprofloxacin (CPX)—Collagen (C) Sponges g CPX/g C	Correlation Coefficient, R			Kinetic Constant, k (1/min^n^)	Release Exponent, n	Drug Released (%)
	Higuchi Model	Zero-Order Model	Power Law Model			
0.5	0.9448	0.7961	0.9762	0.084	0.33	81.63
0.75	0.9716	0.8502	0.9875	0.044	0.37	61.70
1	0.9572	0.8194	0.9820	0.063	0.35	69.86

**Table 2:** Inhibition diameter of the tested sponges against different pathogens.

Sample	Inhibition Diameter, mm			
	*Staphylococcus aureus*	*Escherichia coli*	*Candida albicans*	*Candida parapsilopsis*
100 μg ciprofloxacin/mL	35.0 ± 1.75	0	0	0
1000 μg ciprofloxacin/mL	42.5 ± 2.12	44.0 ± 2.20	0	0
Collagen sponge (C)	10.5 ± 0.52	0	0	0
Ciprofloxacin–collagen sponge (0.5 g ciprofloxacin/g collagen) (C3)	37.0 ± 1.85	42.0 ± 2.10	0	0
Ciprofloxacin–collagen sponge (0.75 g ciprofloxacin/g collagen) (C4)	40.0 ± 2.00	42.0 ± 2.10	0	0
Ciprofloxacin–collagen sponge (1 g ciprofloxacin/g collagen) (C5)	38.5 ± 1.92	44.5 ± 2.22	0	0

**Table 3:** HUVEC proliferation at different time exposure to ciprofloxacin-based materials.

Sample	PI		
	6 h	24 h	48 h
100 μg ciprofloxacin/mL	1.17 ± 0.0234	1.21 ± 0.0182	1.08 ± 0.0216
Collagen sponge	1.34 ± 0.0268	1.29 ± 0.0194	1.23 ± 0.0246
Ciprofloxacin-collagen sponge (0.5 g ciprofloxacin/g collagen)	1.46 ± 0.0292	1.48 ± 0.0222	0.94 ± 0.0188
Ciprofloxacin-collagen sponge (0.75 g ciprofloxacin/g collagen)	1.56 ± 0.0312	1.36 ± 0.0204	1.04 ± 0.208
Ciprofloxacin-collagen sponge (1 g ciprofloxacin/g collagen)	1.24 ± 0.0248	1.16 ± 0.0174	0.77 ± 0.0154
